# Gratitude Predicts Meaning in Life in Family Caregivers of Persons with Alzheimer’s Disease

**DOI:** 10.3390/geriatrics9030072

**Published:** 2024-05-30

**Authors:** Jocelyn Shealy McGee, Edward C. Polson, Dennis R. Myers, Angela M. McClellan, Weiming Ke, Holly Carlson Zhao, Rebecca Meraz

**Affiliations:** 1 Diana R. Garland School of Social Work, Baylor University, Waco, TX 76701, USA; clay_polson@baylor.edu (E.C.P.); dennis_myers@baylor.edu (D.R.M.); ammcclel@central.uh.edu (A.M.M.); 2 Louise Herrington School of Nursing, Baylor University, Dallas, TX 75246, USA; weiming_ke@baylor.edu (W.K.); rebecca_meraz@baylor.edu (R.M.); 3 Center for Optimal Brain Health, Houston, TX 77057, USA; hcarlson@cfobh.com

**Keywords:** Alzheimer’s disease, burden, coping, dementia, family caregiving, gratitude, meaning, well-being

## Abstract

Gratitude is a well-known and researched internal positive psychological resource. Empirical data, however, on the association between gratitude, meaning in life, and burden in family caregivers of persons with Alzheimer’s disease is scant. The aims of this study were to (1) investigate the relationships among these variables in a sample of family caregivers of persons with Alzheimer’s; and (2) determine if gratitude mediates the effects of perceived burden on meaning in life in this population. One-hundred and twenty-six adult family caregivers, most of whom were an intimate partner or adult child of a person with Alzheimer’s, completed the Gratitude Questionnaire-Six Item, the Meaning in Life Questionnaire, the Zarit Burden Inventory, and other relevant measures. A series of OLS regression models, guided by the caregiver stress process model, were conducted. These analyses demonstrated that gratitude was a predictor of the presence of meaning in life among the caregivers in this study even when other key variables were considered. Furthermore, analyses revealed that gratitude fully mediated the effects of caregiver burden on the presence of meaning in life in this sample. Thus, clinicians should consider gratitude as an important internal resource for cultivating meaning in life in this population, especially when caregiver burden is present. Gratitude-bolstering clinical interventions should be further developed and tested as both stand-alone and complimentary additions to empirically supported psychoeducational approaches for supporting health and well-being in this population.

## 1. Introduction

It is well known that primary family caregivers of persons with Alzheimer’s disease and other age-related progressive neurocognitive disorders can experience heightened stress which can potentially negatively impact their health and well-being [1]. However, less is known about how internal positive psychological resources, such as gratitude on the part of family caregivers, contribute to reducing burden and enhancing meaning in life in this population within the realities of their lived experiences. As background for the current study, an overview of the impacts of caregiver burden is provided. Additionally, conceptualizations of the constructs of gratitude and meaning in life are reviewed along with recent research on these constructs in family caregivers. To our knowledge, this is among the first studies to empirically examine the relationships between caregiver burden, gratitude, and meaning in life in family caregivers of persons with Alzheimer’s. We hope that this study stimulates additional research on developing clinical interventions for this distinct population that leads to enhancing their health and well-being.

### 1.1. Caregiver Burden

In general, caregiver burden can be understood as the strain or load carried by a family caregiver of a person with a chronic or terminal health condition [2]. Higher burden levels among family caregivers of persons with Alzheimer’s and other forms of progressive age-related neurocognitive diseases are associated with untoward health outcomes for caregivers and also care recipients [1]. Indeed, burdened caregivers may find it more difficult to meet the care needs of their loved ones [3] potentially leading to increased loneliness [4] and higher mortality rates [5] in persons with Alzheimer’s, although the mechanisms for these outcomes are not well understood.

A range of factors may impact burden perceptions among family caregivers of persons with Alzheimer’s [6] such as being a young-old versus old-old caregiver [7], identifying as female versus male [8], and identifying as sexual or gender-nonconforming versus cis-gendered [9]. There is evidence that wives experience more burden than husbands [10] and adult children experience more burden than spousal caregivers [11]. There is some evidence that caregivers who identify as Black or African American tend to have worse physical wellbeing outcomes as compared to their White counterparts, even when their overall psychological well-being may be similar or better [12]. Conversely, having greater social support [13], drawing from one’s religious and spiritual beliefs and practices [14,15,16,17], and hope [18] can reduce the degree of burden experienced in family caregivers of persons with Alzheimer’s.

### 1.2. Conceptualizations of Gratitude and Research on Gratitude in Dementia Family Caregivers

Gratitude is a positive psychological resource that has been conceptualized as a set of emotions, thoughts, and actions that can be activated and sustained by the acceptance and recognition of unearned or unanticipated benefits that promote well-being, strengthen relationships, and support personal goals [19]. Gratitude has also been described as an overall life orientation [20,21] with some individuals having what McCullough and colleagues describe as a natural tendency towards gratitude or a *grateful disposition* [22].

Gratitude has also been framed as relationship-based human strength involving a shared experience [23] between a benefactor and a beneficiary, resulting in a desired outcome [24] which leads to appreciation [25]. There is evidence that the intensity of gratitude experienced by a beneficiary may be proportional to their perceptions of sacrificial cost, intentionality, and freedom of obligation from their benefactor [26].

Recent research on family caregivers of persons with dementia, who are high in trait gratitude, suggest that they experience lower levels of burden than those who are low in trait gratitude [27]. Family caregivers of persons with dementia who have high levels of gratitude also tend to have greater psychological resilience [28] and empathy [29] than those with lower levels of gratitude. In a recent qualitative study, several themes emerged around the nature of how gratitude is experienced and manifest among caregivers of persons with early-stage Alzheimer’s [30]. Overall, gratitude may be an important resource for cultivating better outcomes in this population.

### 1.3. Conceptualizations of Meaning in Life and Research on Meaning in Life in Dementia Family Caregivers

Victor Frankl [31] theorized that meaning making is a core human motivation. Steger [32], building upon Frankl’s existential perspective, conceptualized meaning in life as having two dimensions: the presence of meaning in life and the search for meaning in life. These two aspects of meaning in life appear to be distinct [33,34]. For example, a person with a strong degree of presence of meaning in life would see themselves as having an overall purpose, a mission, or a vision for their life [32]. In contrast, people searching for meaning in life are engaging in a dynamic and active effort to find and understand their meaning, significance, and purpose [35].

Among family caregivers of persons with dementia, the presence of meaning in life has been associated with greater personal well-being [36], better mental health [37], believing that their sacrifices have been worthwhile [38], and a reduced sense that the caregiving role is burdensome [37]. However, little is known about the mechanisms that lead to the presence of meaning in life in this unique population.

### 1.4. Theoretical Framework and Current Study Aims

The caregiver stress process model [39,40] which addresses the complexity of the caregiving experience, was the theoretical model utilized for variable selection and specification in this study. In this model, stress is categorized as primary or secondary. Primary stress involves the objective demands of caregiving (i.e., the amount of physical care required, the degree of cognitive impairment in the care recipient, etc.). Secondary stress accounts for sources of strain not directly related, but consequential, to the caregiving role such as the sociodemographic profiles of caregivers and care recipients and other contextual factors. In this model, gratitude would be considered a potentially positive internal psychosocial resource which may directly or indirectly contribute to reducing the untoward effects of caregiver stress which may result in a sense of burden. External resources (i.e., social support) may also mediate or moderate relationships among contextual factors, stressors, and caregiver outcomes in this model. The presence of meaning in life, among caregivers in this study, was considered a positive outcome, with gratitude serving as a possible resource for overcoming stress and burden.

The aims of the current study were to (1) investigate the relationships among gratitude, caregiver burden, and meaning in life (presence and search) in a sample of family caregivers of persons with Alzheimer’s disease; and (2) determine if gratitude is a mediator between caregiver burden and the two aspects of meaning in life in this sample.

## 2. Materials and Methods

### 2.1. Study Design and Participants

A cross-sectional quantitative design was utilized in the current study with a convenience sampling strategy. The study was open to adults age of 21 years or older who self-identified as the primary family caregiver of a person diagnosed with probable Alzheimer’s disease and were proficient in the English language. Exclusion criteria were significant cognitive impairment or untreated serious mental illness in caregiver participants.

Flyers about the study were distributed by staff at local medical and community organizations who served persons with Alzheimer’s and their family members in a large metropolitan area in the southwestern part of the USA. An in-person appointment was scheduled for informed consent after telephone screening was conducted for eligibility. Once informed consent was obtained, participants were invited to complete a packet of self-report measures in a private office space free of distractions by a research assistant. It took on average 45 min for participants to complete the packet of measures. Participants did not receive any incentives to participate in the study.

Sample size was based on a priori power analysis to find significance with a desired power of 0.80, an α-level at 0.05, and a medium effect size of 0.15 (f2) as recommended by Cohen [41]. A sample size of at least 97 participants was needed for multiple linear regression analysis with 6 predictor variables. Thus, the obtained sample size of 126 participants was adequate for the analyses in the current study.

### 2.2. Variables and Measures

Standardized measures were used to assess the variables of gratitude, caregiver burden, and meaning in life (search and presence). The variables of intensity of care, social support, and demographic/background information were assessed with investigator-developed measures. Variables and corresponding measures are described below.

#### 2.2.1. Outcome Variables

Meaning in Life. The Meaning in Life Questionnaire (MLQ) [42] is a 10-item self-report instrument with 2 subscales measuring different aspects of meaning in life: (1) presence of meaning in life (how much a person believes that their life has meaning in it); and (2) search for meaning in life (how a person is striving to find meaning in their life). This measure uses a 7-point Likert scale ranging from 0 (Absolutely Untrue) to 6 (Absolutely True) for each item. Good internal consistency, convergent validity, and discriminant validity have been documented for the MLQ for both subscales [42]. Adequate reliability was demonstrated for both the MLQ Presence (Cronbach α = 0.81) and the MLQ Search (Cronbach α = 0.89). subscales in the current study.

#### 2.2.2. Predictor Variables

Gratitude. The Gratitude Questionnaire-Six Item (GQ-6) [22] is a self-report instrument for measuring gratitude. This instrument consists of six items (e.g., “I have so much to be thankful for.”) on a 7-point Likert scale, ranging from 0 (Strongly Disagree) to 6 (Strongly Agree). The GQ-6 has good reliability, with alphas ranging from 0.82 and 0.87 [22]. Adequate reliability was demonstrated in the current study (Cronbach α = 0.81).

Caregiver Burden. The Zarit Burden Inventory [43] has 22 items. Response options are presented on a 5-point Likert scale, ranging from 0 (Never) to 4 (Nearly Always). This measure has demonstrated good internal consistency in other studies (Cronbach α = 0.92; [44]. Adequate reliability was demonstrated in the current study (Cronbach α = 0.94).

Caregiver Stress. An intensity of care index was developed by the investigators for assessing objective stress in this sample. This index was constructed by summing caregivers’ responses to two descriptive survey items: (1) the average number of hours per day spent caregiving (0 to 24 h per day); and (2) the number of people cared for (responses could range from 0 to 5). Scores on this additive index could range from 0 to 11, with higher scores reflecting a greater intensity of care responsibilities.

Social Support. The social support measure utilized in this study was composed of 4 items using a 5-point Likert scale, ranging from 1 (Strongly Agree) to 5 (Strongly Disagree). The higher the score on this measure, the greater the perceived social support. Although this social support measure is not standardized, it has been utilized as a part of the clinical protocol for the Alzheimer’s Disease and Memory Disorders Center at Baylor College of Medicine at intake and at every 6-month visit. Therefore, the accuracy of this measure has been verified by extensive clinical use. In the current study, this measure demonstrated adequate internal consistency (Cronbach α = 0.74).

### 2.3. Data Analysis

Data were analyzed using IBM SPSS Statistics (Version 27). To examine the aims of the study, descriptive statistics were calculated for each participant’s demographic and background variables. These were summarized as means and standard deviation for continuous variables and as counts and percentages for discrete variables. Second, correlations between the outcome variables (meaning in life presence and meaning in life search); the measures for objective, subjective, and secondary stressors; the measures for external and internal resources; and demographic/background variables were calculated. Diagnostic analyses were also conducted to assess for multicollinearity between the independent variables in our regression models. Variance inflation factor (VIF) scores were all below 2.0, indicating multicollinearity was not a significant issue. Third, a series of ordinary least squares (OLS) regression models was developed to determine whether gratitude independently contributed to the explanation of variance in the criterion variables when controlling for other key variables (i.e., gender, age, social support). Finally, a series of mediation models was conducted to test whether gratitude had a mediating effect on the relationship between caregiver burden and meaning in life. Multiple linear regression models were used to estimate and test the paths of c_1_, c_2_, a_1_, a_2_, b, c_1_^’, and c_2_^’. The criterion for statistical significance was set at *p <* 0.05 for all analyses.

## 3. Results

### 3.1. Participant Demographics

One-hundred and fifty-five family caregivers of persons with Alzheimer’s participated in this study. However, for the current analyses, we have restricted the sample size to 126 to account for missing data. Their average age was 64.65 (SD = 11.22), with most caregivers identifying as female (69.8.%, n = 88), White (96.8%, n = 122), and as the intimate partner (i.e., spouse) of their loved one with Alzheimer’s (65.9%, n *=* 83), with a smaller number of adult child caregivers (23%, n *=* 29). See Table 1.

### 3.2. Descriptive and Correlational Data

For the gratitude measure, participants’ scores ranged from 17 to 36 (*M* = 30.93, SD = 5.04). For meaning in life, participants’ scores on the presence subscale ranged from 8 to 30 (*M* = 22.78, SD = 5.30) and on the search subscale from 0 to 30 (*M* = 13.53, SD = 8.03). For caregiver burden, participants’ scores ranged from 5 to 83 (*M* = 30.92, SD = 15.25). The intensity of care index ranged from 0 to 8 (*M* = 3.29, SD = 2.31). For social support, participants’ scores ranged from 10 to 20 (*M* = 17.14, SD = 2.54). See Table 2 for descriptive data.

Gratitude was positively associated with social support, r(124) = 0.461, *p* < 0.001, and negatively associated with caregiver burden, r(124) = −0.264, *p* < 0.001. No significant correlation between gratitude and intensity of care, caregiver age, or caregiver gender was found.

The presence of meaning in life scale was positively associated with social support, r(124) = 0.415, *p* < 0.001, and negatively associated with caregiver burden, r(124) = −0.260, *p* < 0.01. No significant correlation between presence of meaning in life and intensity of care, caregiver age, or gender were found.

In contrast, the search for meaning in life was positively associated with caregiver burden, r(124) = 0.214, *p* < 0.05 and negatively associated with social support, r(124) = −0.186, *p* < 0.05. No significant correlation was found between the search for meaning in life and intensity of care, caregiver age, or caregiver gender. There was no significant association between the presence and search for meaning in life subscales.

Correlations between gratitude and the two dimensions of meaning in life were measured. Gratitude was positively correlated with the presence of meaning in life subscale, r(124) = 0.619, *p* < 0.001. There was a small negative correlation between gratitude and the search for meaning in life subscale r(124) = −0.180, *p* < 0.05. Correlational data are presented in Table 3.

### 3.3. Independent Effects of Gratitude on Meaning in Life Presence and Search

To address the first aim of this study, a series of stepwise hierarchical regression models was developed. Results are presented in Table 4 for the presence of meaning in life subscale and Table 5 for the search for meaning in life subscale. In each table, the first model served as a base model and included measures of primary objective stress (intensity of care), primary subjective stress (caregiver burden), secondary stress (caregiver age, caregiver gender) and an external resource (social support). The second model in each table incorporated the internal caregiver resource and the primary dependent variable of interest, gratitude. To determine whether the inclusion of gratitude had a significant impact on the explained variation, the change in *R*^2^ (*∆R*^2^) between the base models and second models was tested.

The results of the base model indicated that the objective stressor, intensity of care (*β* = 0.194, *p* < 0.05); the secondary stressor, caregiver gender (*β* = 0.181, *p* < 0.05); and the external resource, social support (*β* = 0.348, *p* < 0.001) were all significantly and positively related to meaning in life—presence. Identifying as female, having higher levels of social support, and reporting greater care responsibilities were all correlated with higher levels of meaning in life—presence. In contrast, caregiver burden (*β* = −0.253, *p* < 0.01) was negatively related to the presence of meaning in life. As burden rose, scores on the criterion variable fell. Standardized betas indicated that the strongest predictor in the base model was social support.

In Model 2, we added the variable for gratitude. Regression results indicated that gratitude had a strong, significant, and positive relationship with the presence of meaning in life (*β* = 0.491, *p* < 0.001). Standardized betas revealed that gratitude was the strongest predictor in Model 2, and that when it was included in the model, none of the remaining variables retained their significance. Moreover, adding gratitude to the model significantly improved the overall fit of the model. There was an increase in *R*^2^ from 0.27 to 0.44, indicating a 60.95% increase in variance explained (*∆R*^2^ = 0.167, *p* < 0.001).

In Table 5, the results of the base model revealed that intensity of care (*β* = −0.186, *p* < 0.05) and caregiver gender (*β* = −0.209, *p* < 0.05) were negatively associated with the search for meaning in life. More specifically, identifying as female and reporting more care responsibilities were related to lower scores on the meaning in life search subscale. In contrast, burden (*β* = 0.277, *p* < 0.01) was significantly and positively related to the meaning in life search scale. As burden increased, so did scores on the search for meaning in life subscale. Interestingly, the inclusion of gratitude in Model 2 had no significant impact on the model’s overall fit. Participants’ gratitude scores were not significantly related to the search for meaning in life subscale, and there was no change in the significance of the remaining variables. The strongest predictor of meaning in life—search in both Model 1 and Model 2 was burden.

### 3.4. Gratitude Mediation Effects on Caregiver Burden and Meaning in Life Relationship

**Figure 1:**
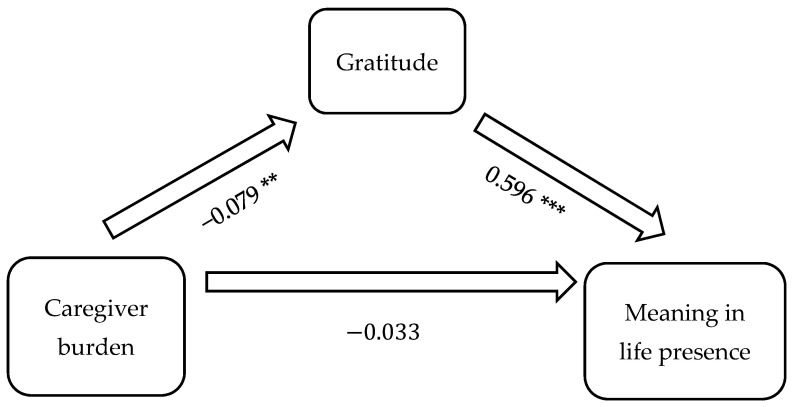
Gratitude mediates caregiver burden on meaning in life in family caregivers of persons with Alzheimer’s disease. Note: ** *p* < 0.05, *** *p* < 0.01.

Figure 1 illustrates the mediation model including the causal variable (burden), outcome variable (the presence of meaning in life), and proposed mediator (gratitude). The results revealed that the direct effect of caregiver burden on meaning in life was −0.033, while the indirect effect, mediated through gratitude, was *ab* = (−0.079)(0.596) = −0.047. The indirect effect accounted for 58.75% of the total effect. The results of the Sobel test revealed that the mediation effect of gratitude on the relationship between caregiver burden and the presence of meaning in life was statistically significant (*p* = 0.006). There was no evidence of a moderation effect for gratitude on the presence of meaning in life.

## 4. Discussion

In the current study, we examined relationships between gratitude, caregiver burden, and meaning in life (presence and search). Our findings suggest an interplay between these variables which may be important to the overall health and well-being of caregivers of persons with Alzheimer’s disease and other age-related progressive neurocognitive conditions. This study is the first, to our knowledge, to examine the influence of gratitude on caregivers’ sense of meaning in life.

Our findings provide evidence that higher levels of gratitude among family caregivers of persons with Alzheimer’s is significantly associated with the greater presence of meaning in their lives even when key stressors and demographic variables were taken into consideration. Similar to our findings, previous work by Nah and colleagues [28] demonstrated that greater perceived gratitude among caregivers of adults with chronic illness or disability was associated with better psychological wellbeing. They also found that gratitude buffered the association between role overload, a concept related to caregiver burden and psychological wellbeing.

Of import, gratitude mediated the relationship between caregiver burden and the presence of meaning in life in the caregivers in this study. This finding is consistent with studies documenting the mediation effects of gratitude on meaning in life and aging [45] and time perspective [46]. The grateful response of the care recipient has been a suggested reason for the mediating effect of gratitude between caregiver burden and meaning in life. In a study of family members who cared for an older relative, Otobe et al. [29] found that caregivers who received more expressions of gratitude from their loved one had lower rates of caregiver burden.

Algoe’s [47] find-remind-and-bind theory of gratitude emphasizes gratitude as a bonder of human relationships and an impetus for continuous spirals of mutuality. This was echoed in a recent qualitative study in which caregivers of persons with early-stage Alzheimer’s disease expressed their gratitude for the opportunity to provide and also receive care from their loved ones [30]. While the care recipient’s expressions of gratitude may be a source of grateful responses among caregivers of persons with dementia, this factor is limited by the relational realities of the dementia care trajectory. With time, the care recipient’s capacity to express gratitude may diminish, and the caregiver may not be recognized by the person living with dementia. Future qualitative studies may provide the most effective method for identifying alternative stimuli for the caregiver’s grateful response and enriched meaning. Overall, additional research is warranted on how gratitude among family caregivers of persons with dementia may impact the meanings they attribute to the caregiving role itself.

A possible interpretation for our discovery that gratitude strongly predicts meaning in life presence, even in the context of burden, is that gratitude functions as an internal positive psychological asset. The research literature suggests that internal psychological resources such hope [18] and practicing positive spiritual techniques [14,15,16,17,48] impact meaning in life and one’s sense of purpose in this population [48]. Gratitude’s beneficial influence on meaning in life may also enhance a caregiver’s coping skills, buffering the impact of caregiving challenges [37].

A sense of meaning in life can enable people to contribute to the capacity to deal with physical and psychological pain, create meaning from these experiences, and reactivate their sense of well-being [49]. Family caregivers who practice gratitude frequently may be more aware of the positive aspects of caregiving which could lead to enhanced emotion-focused coping and lower levels of distress [27].

Given the relationship between gratitude and the presence of meaning in life in the current study, it is apparent that even small incremental increases in gratitude can positively influence a caregiver’s life. A better understanding of how to further cultivate and activate this positive psychological resource among family caregivers may contribute to reducing the consequences of burden and positively impact the caregiving experience.

Wood and colleagues’ Life Orientation Model [20] which has been applied to general populations, provides insight into possible mechanisms between dispositional gratitude and meaning in life. First, according to this model, persons in general populations with higher levels of dispositional gratitude are more likely to perceive their lived experiences as worthy of gratitude than those with lower levels of dispositional gratitude [20]. We suggest that family caregivers who have higher levels of dispositional gratitude may be more likely to notice and reflect on their overall lived experience as gratitude-worthy and thus meaningful. 

Additionally, we suggest that some family caregivers may be more inclined than others to notice with presence, awareness, and gratitude the day-to-day happenings in their lives within their caregiving role as well as the other aspects of their lives. This capacity to notice with gratitude and attribute meaning to one’s life as a whole and each aspect of daily living is what we could be considered what we refer to as *mindful caregiving* and cultivated through various techniques and practiced.

Surprisingly, gratitude did not contribute to explaining variation in caregivers’ searching for meaning in this study. Whether or not gratitude was included in the model, caregiver burden was the strongest predictor of searching for meaning in life. A possible explanation of this finding is that for caregivers who were seeking but not experiencing a present and viable sense of meaning, gratitude may not have been as readily available to activate meaning or reduce perceived burden. Perhaps this finding highlights the importance of developing gratitude-oriented mindfulness interventions aimed at drawing caregivers’ attention to current blessings while also acknowledging their significant life challenges, thus leading to greater presence of meaning in life.

## 5. Study Limitations

First, we acknowledge the findings of this study are not generalizable to the experience of caregivers of persons living with dementia internationally or even within the United States. Rather, our multivariate analyses provided insight into the relationship between gratitude and meaning in life in a mostly White sample of caregivers who primarily self-identified as women in a large metropolitan area in the United States. Second, given that the study was based on self-reporting, a social desirability bias may have been present. Likewise, participants were aware that the study was focused on positive psychological aspects of caregiving, which may have limited participation to caregivers who were interested in this topic. A longitudinal study is also necessary for understanding how gratitude informs meaning in life among caregivers over time and as dementia progresses. Further study of the benefactor and beneficiary relationship is needed, as well as the reciprocal and iterative aspects of caregiver gratitude. Likewise, qualitative and mixed-methods approaches may serve to reveal a greater depth of understanding.

## 6. Recommendations

Our findings suggest that gratitude is a construct worthy of consideration by researchers and those who serve family caregivers and their loved ones living with dementia as a way to enhance meaning and purpose in their lives. Three recommendations flow from the evidence provided in this study.

### 6.1. Develop and Test Evidence-Informed Caregiver Gratitude Assessment and Intervention Protocols

Gratitude mediated perceived burden among caregivers leading to a greater sense of meaning in their lives in this study. Therefore, we recommend including measures of these constructs in clinical assessment and research protocols for enhancing caregiver health and well-being. Including measures of positive psychological constructs, such as gratitude and meaning in life, may increase clinicians’ capacities to improve care planning and assess caregiver progress over time when receiving clinical intervention(s). We advocate for the use of brief standardized measures, with adequate psychometric properties, rather than the use of lengthy protocols. This approach may allow for clinicians to gain the information they need to effectively serve caregivers while reducing the amount of effort that caregivers must out forth during clinical assessment.

The mediation finding also supports further development and clinical trial validation of gratitude interventions for this population, promoting the potential capacity of caregiver gratitude to recognize, develop, and sustain meaning making. There are several recent reviews and metanalyses on gratitude interventions [50,51,52] which may guide the development of caregiver-specific gratitude-oriented interventions.

### 6.2. Deepen Understanding of the Caregiver Gratitude and Meaning Relationship

Nomothetic studies, such as this one, are not able to tap into the interior and day-to-day relational experiences of caregivers and their loved ones with Alzheimer’s. Increased engagement with the narratives of caregivers will illuminate the complexities of the caregiver-care receiver relationship and clarify sources of caregiver gratitude and meaning. Mixed-method approaches testing the conceptual frames identified in the current research on gratitude could provide validation and in-depth understanding of the applicability of these constructs in explaining meaning-making among caregivers with persons with dementia.

### 6.3. Focus on Cultural, Ethnic, Gender, and Spiritual Diversity

Expressions of human gratitude are as diverse as the array of international, national, and community cultures. There is a need to take into consideration diverse cultural, spiritual, and philosophical understandings of gratitude and meaning in life. There is a particular notable dearth of published research on First Nation or indigenous caregivers—as well as immigrants and displaced persons. Effective and ethical future research will require further attention to diverse caregivers within their unique cultural, ethnic, and/or religious/spiritual contexts.

## 7. Conclusions

Guided by the caregiver stress process model [39,40], for variable specification, and extant research on the relationships between gratitude, caregiver burden, and meaning in life, this study provides evidence that gratitude is a key factor in caregivers’ sense of the presence of meaning in life, even within the context of perceived burden. Indeed, having a grateful life orientation mediated the relationship between perceived caregiver burden and the presence of meaning in life in the context of caring for persons living with Alzheimer’s in the participants in the current study. We provide implications of our findings intended to inform researchers and professionals seeking to increase caregiving meaning and reduce burden by testing, applying, and evaluating evidence-informed, culturally sensitive assessments and interventions intended to strengthen caregiver gratitude and improve outcomes for those for whom they care.

## Figures and Tables

**Table 1:** Demographic information for sample.

	Mean (SD)	n (%)
Caregiver age in years (range: 22–87)	64.65 (11.22)	
Caregiver gender		
Male		38 (30.2)
Female		88 (69.8)
Caregiver education		
High school or equivalent		10 (8.0)
Undergraduate		82 (65.6)
Graduate		31 (24.8)
Other		2 (1.6)
Caregiver ethnicity		
Hispanic or Latino		4 (3.2)
Not Hispanic or Latino		122 (96.8)
Caregiver race		
Person of Color		3 (2.7)
White		122 (96.8)
Unspecified		1 (0.8)
Relationship to person with dementia		
Spouse/intimate partner		83 (65.9)
Adult child/grandchild		29 (23.0)
Other		14 (11.1)

**Table 2:** Descriptive statistics for measures.

	N	Range	Mean	SD	Cronbach’s Alpha
**Outcome**					
Meaning in life presence	126	8–30	22.78	5.30	0.81
Meaning in life search	126	0–30	13.53	8.03	0.89
**Primary Stressors**					
Objective (intensity of care) *	126	0–8	3.29	2.31	N/A
Subjective (burden)	126	5–83	30.92	15.25	0.94
**Secondary Stressors**					
Caregiver age in years	126	22–87	64.65	11.22	N/A
Caregiver gender	126	1–2	1.70	0.46	N/A
**Resources**					
Social support	126	10–20	17.14	2.54	0.74
Gratitude	126	17–36	30.93	5.04	0.81

* Intensity of care is an additive index representing the sum of hours spent providing care for the persons with Alzheimer’s and the total number of people cared for. Therefore, an alpha was not calculated for this index.

**Table 3:** Correlation matrix for variables.

Variable	1	2	3	4	5	6	7	8
1. Meaning in life presence	-							
2. Meaning in life search	−0.210 *	-						
3. Intensity of care	0.129	−0.131	-					
4. Caregiver burden	−0.260 **	0.214 *	0.243 **	-				
5. Social support	0.415 ***	−0.186 *	−0.076	−0.248 **	-			
6. Caregiver age	0.124	−0.089	0.133	−0.136	−0.010	-		
7. Caregiver gender	0.139	−0.142	0.052	0.262 **	0.110	−0.220 *	-	
8. Gratitude	0.619 ***	−0.180 *	0.131	−0.264 **	0.461 ***	−0.02	0.153	-

*Note*: * *p* < 0.05. ** *p* < 0.01. *** *p* < 0.001.

**Table 4:** Standardized OLS coefficients predicting meaning in life presence.

	Model 1	Model 2
Intensity of care	0.194 *	0.084
Caregiver burden	−0.253 **	−0.121
Caregiver age	0.108	0.131
Caregiver gender	0.181 *	0.104
Social support	0.348 ***	0.155
Gratitude		0.491 ***
*Intercept*	4.769	−4.096
*^a^ F*	9.057 ***	15.666 ***
*^b^ R* ^2^	0.27	0.44
*^c^ ∆R* ^2^		0.17 ***
*N*	126	126

Note: * *p* < 0.05, ** *p* < 0.01, *** *p* < 0.001. *^a^* Test statistics for the *F*-test. *^b^* R-squared coefficient of determination. *^c^* R-squared change.

**Table 5:** Standardized OLS coefficients predicting meaning in life search.

	Model 1	Model 2
Intensity of care	−0.186 *	−0.186 *
Caregiver burden	0.277 **	0.277 **
Caregiver age	−0.073	−0.073
Caregiver gender	−0.209 *	−0.209 *
Social support	−0.109	−0.108
Gratitude		−0.002
*Intercept*	26.64	26.69
*^a^ F*	3.916 **	3.236 **
*^b^ R* ^2^	0.14	0.14
*^c^ ∆R* ^2^		0.00
*N*	126	126

Note: * *p* < 0.05, ** *p* < 0.01. *^a^* Test statistics for the *F*-test. *^b^* R-squared coefficient of determination. *^c^* R-squared change.

## Data Availability

The data presented in this study are not publicly available for the purposes of the privacy and confidentiality of research participants.
